# The Role of the m^6^A RNA Methyltransferase METTL16 in Gene Expression and SAM Homeostasis

**DOI:** 10.3390/genes13122312

**Published:** 2022-12-08

**Authors:** Jacqueline E. Mermoud

**Affiliations:** Institute of Molecular Biology and Tumor Research, Philipps University Marburg, 35043 Marburg, Germany; mermoud@imt.uni-marburg.de; Tel.: +49-6421-2826349

**Keywords:** N6-methyladenosine, chromatin, DNA methylation, histone methylation, METTL16, epitranscriptome, epigenetics, metabolism, S-adenosyl-methionine, MAT2A

## Abstract

The RNA methylation of adenosine at the N6-position (m^6^A) has attracted significant attention because of its abundance and dynamic nature. It accounts for more than 80% of all RNA modifications present in bacteria and eukaryotes and regulates crucial aspects of RNA biology and gene expression in numerous biological processes. The majority of m^6^A found in mammals is deposited by a multicomponent complex formed between methyltransferase-like (METTL) proteins METTL3 and METTL14. In the last few years, the list of m^6^A writers has grown, resulting in an expansion of our understanding of the importance of m^6^A and the methylation machinery. The characterization of the less familiar family member METTL16 has uncovered a new function of the m^6^A methylation apparatus, namely the fine-tuning of the cellular levels of the major methyl donor S-adenosylmethionine (SAM). METTL16 achieves this by adjusting the levels of the enzyme that synthesizes SAM in direct response to fluctuations in the SAM availability. This review summarizes recent progress made in understanding how METTL16 can sense and relay metabolic information and considers the wider implications. A brief survey highlights similarities and differences between METTL16 and the better-known METTL3/14 complex, followed by a discussion of the target specificity, modes of action and potential roles of METTL16.

## 1. m^6^A RNA Methylation

The regulation of gene expression through reversible chemical modifications of RNA, DNA and histone proteins is of paramount importance for normal development and differentiation. One of the most prominent modifications detected in mRNA is N^6^-methyladenosine [1,2]. Many recent studies have underscored that m^6^A methylation is also present in different types of non-coding RNAs [2,3,4,5,6] and plays important roles in diverse cellular processes, including stem cell differentiation and neurogenesis [2,7,8]. The dysregulation of m^6^A modification has been associated with aberrant animal development and several human diseases, notably cancer [8,9,10,11]. The functional consequences of m^6^A methylation are wide-ranging since m^6^A methylation has been linked to almost every step in RNA homeostasis. Effects on the RNA structure, RNA stability or effector protein binding by m^6^A in turn impact processes such as pre-mRNA splicing, mRNA export, translation initiation and chromatin activity, to name just a few [8,12,13,14,15,16].

Given the potential significance of RNA m^6^A modification in human health and disease, efforts of the past few years have focused on the identification of regulators of this modification. m^6^A methylation is introduced by methyltransferases and can be removed either passively, via the degradation of the modified RNA, or actively, by erasers such as the dioxygenases FTO or ALKBH5 [7,17,18,19]. Resembling the function ascribed to histone modifications, m^6^A modification can cause a structural switch and/or act as a signal for the recruitment of downstream effectors that influence the fate of the target mRNA [20,21,22,23,24]. Many functions of m^6^A are mediated through reader proteins. Some readers, such as YTH-domain-containing family members, can bind directly to m^6^A methylated RNAs. Alternatively, the modulation of the secondary RNA structure can expose or mask RNA-binding motifs recognized by reader proteins, leading to an m^6^A-dependent regulation of RNA maturation and gene expression [7,17,18,19].

## 2. m^6^A Writers

Active methyltransferases transfer a methyl group from the co-factor S-adenosylmethionine (SAM) to the substrate adenosine [25]. Table 1 illustrates that the five m^6^A methyltransferases identified to date are distinct and differ in several important ways. One, they generally target different RNAs and/or sites. For example, ZCCHC4 represents a 28S-RNA-specific methyltransferase [26] whereas METTL3/14 targets mRNAs, non-coding RNAs and primary micro-RNAs [5,20,27]. When a given RNA is modified by more than one writer, this usually involves different sites within the transcript due to distinct substrate and RNA recognition modes of m^6^A writers [25,28].

**Figure 1:**
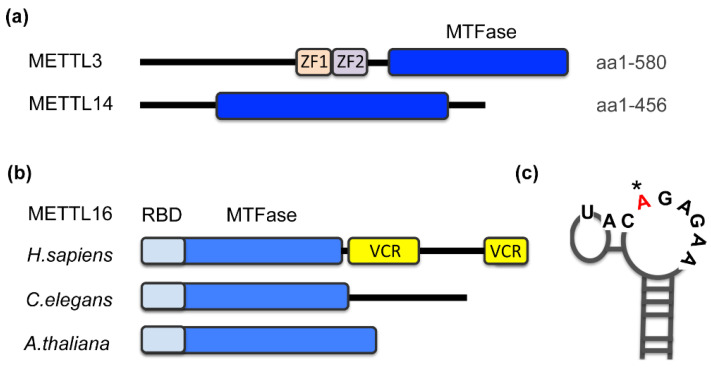
Domain organization of m^6^A methyltransferases (**a**) METTL3, METTL14 and (**b**) METTL16. Schematic indicates domain architecture and function. MTFase, methyltransferase Rossmann fold; ZF, zinc finger domain; RBD, RNA-binding domain, VCR, vertebrate-conserved region. (**b**) METTL16 from selected species that are discussed in this review: human (aa 1-562), worm (METT-10) and *Arabidopsis* (FIONA1). (**c**) Stem-loop structure of a typical METTL16 target site with a specific nonamer sequence; the modified adenosine is highlighted (*). Based on the first hairpin of MAT2A 3′UTR.

Two, different writers display different sequence motif preferences (Table 1), with some enzymes (METTL16; ZCCHC4) favoring a combination of sequence and structural features [28,31,35]. In contrast, METTL3/14 shows little dependency on a particular structure [29]. Three, whereas some RNA methyltransferases act in a complex (e.g., METTL3/14; METTL5) [25,32], others appear to function alone. In the METTL3/14 complex, METTL3 represents the catalytic subunit that binds SAM, whereas the catalytically inactive METTL14 promotes RNA-binding and stimulates methyltransferase activity [25,27,29,37,38]. Accessory factors such as Wilms tumor 1 associating protein WTAP or RNA-binding motif proteins RBM15/15B and others further modulate the METTL3/14 activity and specificity [17,38,39]. In contrast, METTL16 has been found to exist as a monomer or homodimer [28,40,41].

m^6^A methyltransferases harbor a signature motif of class I methyltransferases, the Rossmann fold (Figure 1a,b, MTFase domain), and, in most cases, additional domains that contribute to the regulation of the enzyme [25]. Zinc fingers in METTL3 constitute the RNA recognition domain and cooperate with the MTFase domains for catalysis (Figure 1a) [42]. Deletions of arginine/glycine motifs located in the C-terminus of METTL14 also reduce the RNA-binding affinity of METTL3/14 [43]. METTL16 recognizes its substrates via two domains that bear no resemblance to canonical RNA-binding motifs (Figure 1b). The unique N-terminus is required for RNA-binding and hence catalysis [28,31,41]. In higher eukaryotes, METTL16 additionally contains a C-terminal vertebrate-conserved region (VCR). An arginine-rich sequence within the VCR is critical for substrate binding and methylation. The precise function of this domain is still under investigation [31,44], but it has been shown that the VCR domain enhances the catalytic efficiency by lowering the Km by at least an order of magnitude [44]. In non-vertebrates, it is possible that accessory proteins take on the role of the VCR.

The successful crystallization of METTL16, either of separate protein domains alone or in a complex with RNA, is extremely informative [28,31,41,44]. One outcome was the assignment of a regulatory role to a loop near the SAM-binding pocket that controls SAM-binding and hence methylation efficiency. Auto-inhibition via this so-called K-loop appears to be unique to METTL16 and is not observed in METTL3 [28]. Significant progress has also been made towards an understanding of the molecular basis of RNA- binding. Detailed accounts of these structural insights can be found here [25,45].

## 3. Multifaceted METTL16: Nuclear and Cytoplasmic, Catalytic and Non-Catalytic Roles

Much progress has been made in recent years in identifying RNAs bound by METTL16 [11,44,46,47,48,49,50,51,52,53]. It has emerged that METTL16 interacts with both coding and non-coding RNAs. These include, but are not limited to, small nuclear RNAs such as U6 [47,48], long non-coding RNAs [48,50] such as the cancer-related RNA RAB11B-AS1 [52] and the metastasis—associated lung adenocarcinoma transcript 1 (MALAT1) [46], as well as ribosomal RNA [48,50] and a set of mRNAs [48,53]. Representative examples belonging to different classes of RNAs, namely U6 snRNA, MALAT1 long non-coding RNA and MAT2A mRNA, have been validated as bona fide METTL16 RNA interactors [46,47,48].

One of the first identified and thus far best characterized METTL16 targets is the MAT2A mRNA [31,47,54], which encodes the key enzyme for methyl-donor synthesis in cells. It was subsequently shown that METTL16 is critical for preserving physiological SAM levels as discussed further below. In most cases, however, the function of the observed METTL16-RNA interaction is ambiguous as METTL16 can regulate the fate of its bound RNAs in diverse ways [45,55]. Like METTL3, the METTL16 protein acts in both the nuclear and cytoplasmic compartments, participating in RNA biogenesis, RNA decay and translational control [11,45,50,55]. Current models of how RNA methyltransferases might regulate different steps of gene expression include control through m^6^A modification. Accordingly, methyltransferase-activity-dependent functions of METTL16 have been documented, and have been shown to impact the mRNA stability and splice site choice [47,54,56]. However, one emerging concept is that not all METTL16-bound RNAs are methylated. This is based on the observation that, although METTL16 associates with thousands of RNAs, METTL16-catalyzed m^6^A methylation could be detected in only a small proportion of them [11,48]. The reasons could be, at least in part, technical, leading to an underestimation of methyl-sites. However, considerably more METTL16-bound than METTL16-modified RNAs were observed independent of the particular detection method applied or the type of RNA preparation used (e.g., total RNA versus nascent RNA) [11,48]. This favors a model in which METTL16, besides catalyzing the formation of m^6^A, exhibits significant methylation-independent functions [11,48]. In line with this interpretation, non-catalytic roles for METTL16 in both the nucleus and cytoplasm have been described [11,47,50,57]. For instance, METTL16 acts as a splicing enhancer of the mammalian MAT2A transcript independent of its methyltransferase activity [47]. Moreover, METTL16 prevents DNA-end resection in a methyltransferase-independent manner [57]. Cytosolic METTL16 promotes the efficient translation of thousands of transcripts independent of m^6^A through interactions with eukaryotic translation initiation factor 3a/b [11,50]. Potential contributions of METTL16 and m^6^A to cancer progression have been discussed elsewhere [9,10,11,57,58,59]. To sum up, it is becoming evident that METTL16 is a multifunctional enzyme with nuclear as well as cytoplasmic, catalytic and non-catalytic roles in gene regulation in physiological and pathological settings.

## 4. METTL16 Methylated RNAs

The extent to which METTL16 activity contributes to global m^6^A methylation remains an active area of research. Given that the entire field is young and flourishing, new technologies for high-confidence m^6^A mapping continue to be developed, allowing for the comparison of different datasets derived from m^6^A-antibody immunoprecipitation-based sequencing methods (e.g., m^6^A IP-seq or m^6^A-crosslinking exonuclease sequencing) and antibody-independent methods (e.g., metabolic labeling or small-molecule-based transcriptome editing) [11,47,49,51,53]. Collectively, these studies have pinpointed a few hundred direct m^6^A METTL16 candidate targets, including long ncRNAs, intronic sites and mRNAs associated with the DNA damage response, that await confirmation. A survey of these potential METTL16 methylation targets is provided by [55]. Currently, only two RNAs have been shown to be m^6^A METTL16 targets with any certainty: the mRNA MAT2A and the snRNA U6. Here, studies centered mainly around these two distinct transcripts are discussed to illustrate what is known about the substrate requirements and how METTL16 can regulate the fate of its target RNAs. METTL16 is, to date, the first and only RNA methyltransferase that acts as a metabolic sensor to safeguard SAM homeostasis. Therefore, emphasis will be on the functional significance of METTL16 in the maintenance of physiological SAM levels. Recent results from vertebrates and invertebrates will be reviewed that collectively reveal the principal ways that METTL16 regulates SAM biosynthesis through its catalytic and non-catalytic activities.

How might METTL16 selectively methylate certain transcripts and specific sites? It has been shown that the enzymatic activity of METTL16 is strictly dependent on a specific target sequence in combination with secondary structure features of the RNA (Table 1; Figure 1c). This conclusion is derived from a comprehensive in vitro and in vivo analysis of MAT2A and U6 RNA methylation sites [31,47,54,56] and holds true for independently characterized DNA-repair-related gene transcripts methylated by METTL16 [53]. Although one has to keep the inherent limitations of a very small sample size in mind, the results are exciting, revealing that METTL16 and METTL3/14 enzymes display very distinct substrate specificities. Whereas METTL3/14 exhibits activity towards single-stranded RNA with a “DRm^6^ACH” motif (in which D = A,G or U; R = A or G; H = A,G or U) [1,12,60], METTL16 preferentially methylates a nine-nucleotide consensus sequence UACm^6^AGARAA [28,31,47] (Table 1). However, this nonamer sequence only serves as an effective substrate when it is embedded in the appropriate secondary structure (Figure 1c). For methylation to occur, the target adenosine must be unpaired and flanked by stems, whereby nucleotides adjacent to the bulge influence the methylation efficiency significantly [28,31,56]. This information is frequently used to predict, based on sequence and structural context, which MTFase is responsible for a given m^6^A event in the transcriptome.

Of note, only a fraction of RNAs that contain the sequence consensus motifs for either METTL16 or METTL3/14 have been shown to be methylated [1,19,47]. It is possible that a subset of stimulus-dependent, dynamically regulated sites may have escaped identification [1,60]. Part of the explanation may also lie in the structural pre-requisite for METTL16 activity, since, for productive catalysis, the correct folding of the RNA may be necessary to properly present the target adenine residue to METTL16 [28]. In addition to the sequence and structure, extrinsic determinants are known to play an important role in shaping the m^6^A landscape controlled by METTL3/14 [12,17,61]. This is exemplified by trans-acting factors such as RNA-binding proteins or transcription factors that recruit METTL3/14 to promote the methylation of certain transcripts [12,19,58]. METTL16 is likely to similarly exploit protein co-factors to modulate its specificity and activity, possibly in a developmental or tissue-specific manner. To put this notion to test, several studies set out to purify METTL16 from mammalian cells and mouse tissues, aiming to identify candidate subunits and regulators [31,46,62,63]. Different experimental strategies were pursued to detect stable and transient interactions: either proximity-dependent labeling approaches or affinity purification after the precipitation of endogenous or tagged METTL16 coupled to mass spectrometry. Overall, remarkably few protein interactions were detected and, furthermore, these were largely mediated via RNA rather than direct protein–protein interactions [31,46,62,63]. Therefore, the model to date is that, in contrast to METTL3/14, METTL16 is not part of a stable protein complex and lacks other core subunits. 

Protein–protein interaction networks often provide clues about the biological processes that the bait protein is engaged in. In the case of METTL16, there was little overlap between different datasets, but pre-mRNA splicing factors and U6 biogenesis factors were identified in a subset of the METTL16 interactomes [31,63]. The results of the follow up validation and functional experiments are eagerly awaited. 

To what extent weak transient interactions or post-translational protein modifications (PTMs) are required for directing METTL16 function remain open questions. The phosphorylation of METTL16 induced by DNA damage was reported to result in decreased RNA-binding [57]. Other PTMs could fine-tune the subcellular localization of METTL16 or affect its stability, catalytic activity or protein interaction partners. What is clear is that the outcome of METTL16-deposited methylation can vary from the degradation of the mRNA (e.g., *MAT2A*) [47,54] to upregulation of gene expression (e.g., *Brca2* mRNA) [53]. The relative contribution of canonical m^6^A reader proteins to the implementation of m^6^A signals set by METTL16 is an area yet to be explored. 

Another conundrum is that, while only a small number of direct methylation targets of METTL16 have been confirmed, the depletion of this enzyme results in a genome-wide reduction in m^6^A [47,49]. This finding has important implications. It suggests that METTL16 has a broad impact on the m^6^A landscape through a combination of direct and secondary effects. Indeed, an in-depth motif analysis revealed that the vast majority of the affected sites are in fact METTL3–dependent [47,49]. This likely reflects the crucial role of METTL16 in SAM biosynthesis. Interference with the METTL16 function has been shown to cause SAM reduction [47]. In this way, METTL16 will directly impact methylation events catalyzed by METTL3, which depend on SAM availability [47,49,51]. Going forward, it will be essential to check the dependency of any given m^6^A site on both METTL16 and METTL3 to distinguish direct METTL16 targets. It will also be pertinent to determine the extent to which other RNA-, DNA- and protein methyltansferases that utilize SAM as a co-factor are affected by METTL16.

## 5. Timing of m^6^A Deposition and Its Position within the Transcript

Understanding how, when and where a modification occurs at a particular RNA residue is expected to provide clues to the functional significance of this modification. Efforts to determine the time point during the life cycle of a given RNA when m^6^A is installed by methyltransferases METTL3/14 and METTL16 have benefited from technological advances. One, m^6^A-antibody-based sequencing techniques have been applied not only to the total RNA but also to different cellular RNA fractions, comparing chromatin-associated, nuclear and cytoplasmic RNA pools. Such studies revealed that METTL3 deposits m^6^A co-transcriptionally on polymerase-II-transcribed pre-mRNA and chromosome-associated regulatory RNAs, primarily near terminal exons and within long internal exons of pre-mRNAs and at intergenic regions [64,65,66]. Similarly, METTL16 mainly installs m^6^A onto newly transcribed RNAs, including sites in the vicinity of the start codon, in exons and introns [11,48]. Two, an antibody-independent method (meCLICK-Seq) that relies on the catalytic activity of the enzyme under study likewise identified more m^6^A METTL16 sites in nascent than in mature RNAs [51]. This study revealed that over 75% of METTL16-dependent peaks fall within intronic regions, a much more significant proportion than for METTL3 [51] and than previously reported for METTL16 [11,48]. This difference to other METTL16 studies may reflect the fact that meCLICK-Seq was developed to map m^6^A in low-abundance transcripts derived from intronic and intergenic regions [51]. The authors further validated the intronic m^6^A marks in cell lines that carry deletions in selected intronic regions [51]. How might intronic m^6^A marks installed by METTL16 affect the fate of the transcript? One suggestion put forward is that they are related to intronic polyadenylation [51]. Given that intronic polyadenylation is widespread in cancer and usually leads to the generation of non-coding transcripts or truncated proteins [51], these findings provide a new and exciting direction for future investigations into the consequence of m^6^A marks set by METTL16. 

## 6. METTL16 Methylates a Spliceosomal Component, but Does This Impact Splicing?

METTL16 binds and methylates U6 snRNA [31,47,48], a central component of the spliceosome transcribed by RNA polymerase III. Specifically, METTL16 deposits a single m^6^A methylation in a bulge in the stem of a hairpin structure in human U6 snRNA: the so-called ACAGA box [31,47,48]. This sequence lies in an evolutionarily conserved region important for splicing catalysis since it base-pairs with the 5′ splice site of pre-mRNAs in the first catalytic step of splicing [67,68]. In the past, investigations of the role of this highly conserved sequence motif in U6 snRNA have mainly focused on human and budding yeast, but with the discovery of METTL16 as the enzyme responsible for its methylation, researchers have recently began to look to other species. It was demonstrated that METTL16 orthologs in *C. elegans* and *Arabidopsis* represent the m^6^A writer for U6 snRNA, targeting an adenosine in the same sequence context as in human U6 (UACm^6^AGAGAA) [47,48,56,69,70]. In *S. pombe*, the METTL16 counterpart Mtl16 is responsible for the m^6^A modification at A37 in the ACAGA box; a *mtl16* yeast deletion strain exhibits a loss of U6 snRNA methylation and slower growth rates [71]. Notably, in organisms with a small number of introns such as the budding yeast *S. cerevisiae*, U6 snRNA methylation is missing, and this correlates with the absence of METTL16 [47,71]. In conclusion, METTL16 represents the U6 snRNA methyltransferase that researchers were hunting for since the discovery of m^6^A in human U6 at position A46 over forty years ago [72,73].

What is the consequence of the METTL16 deposition of m^6^A into U6 snRNA? The frequency of this modification is nearly 100% and it occurs during early stages of U6 snRNP biogenesis [48]. U6 snRNA sits at the heart of the spliceosome, where it positions the substrate for the splicing reaction [74]. m^6^A could potentially impact the U6 snRNA function by modulating its stability or its interactions with RNAs and proteins. Methylated U6 snRNA gets incorporated into the U4/U6 snRNP, indicating that this methylation event is functional and possibly structural, and arguing against it being a target for a reader protein [48,74]. Mutations within the U6 snRNA ACAGA motif in yeast are lethal [75]. It is therefore tempting to speculate that the modification of this site impacts pre-mRNA splicing, but empirical evidence for this has proven surprisingly difficult to obtain. Global splicing defects are not readily evident in mammals or plants when METTL16 is depleted [31,53,70]. *mettl16* null mutant mice embryos show little change in splicing patterns [31], but whether a maternal pool of methylated U6 snRNA can compensate for this is unresolved. Alternatively, other active RNA methyltransferases may be able to complement *mettl16* mutants. However, this possibility seems less likely given that there is no evidence that different RNA methyltransferases can substitute for each other in vivo. A contribution of m^6^A U6 snRNA to the fine-tuning of the splice site selection is an attractive concept that requires more thorough investigation [31]. It is plausible that a loss of the U6 methyl-mark triggers subtle differences in the spliceosome assembly or affects the splicing of specific gene transcripts [48]. Indeed, a recent study demonstrates that the loss of U6 snRNA methylation in fission yeast [71] regulates the splicing of a subset of introns, especially those weakly recognized by U5 snRNA, another spliceosomal component [71]. This suggests that m^6^A in U6 can contribute to 5’ splice site recognition in a context-dependent manner. Based on these results, it will be exciting to revisit the question of whether the efficiency of the splicing of particular gene transcripts is affected by U6 snRNA m^6^A in metazoa.

While the biological significance of the METTL16 methylation of U6 snRNA is still debated, particularly for vertebrates, studies in mammals and worms have demonstrated that METTL16 post-transcriptionally regulates the expression of the key enzyme for the production of SAM. The mechanistic detail is different in vertebrates and invertebrates as surveyed below.

## 7. Roles of METTL16 in the Control of SAM Homeostasis

### 7.1. Introduction to SAM Synthetases (MATs)

**Figure 2:**
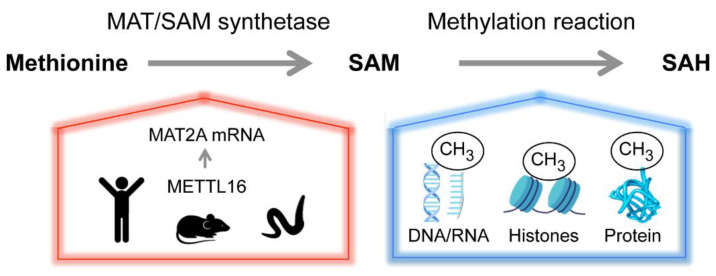
Homeostasis of the versatile methyl donor S-adenosyl-methionine (SAM or AdoMet). Methionine adenosyltransferase (MAT or SAM synthetase) catalyzes the formation of SAM from methionine and ATP. Methyltransferases transfer the methyl group from SAM to a variety of acceptor molecules (e.g., nucleic acids, proteins and lipids). The product, S-adenosylhomocysteine (SAH), is recycled to regenerate methionine but can also act as an allosteric regulator. SAM contributes to several biosynthetic pathways and is central to many cellular functions, including epigenetic regulation, cell growth and maintaining the redox status of the cell. The physiological levels of SAM are tightly controlled. In human, mouse and *C. elegans*, the m^6^A RNA methyltransferase METTL16 senses SAM and controls the abundance of SAM synthetase MAT2A mRNA post-transcriptionally.

The principal methyl-group donor of all cells, SAM, is generated from the amino-acid methionine and adenosine tri-phosphate (ATP) by methionine adenosytransferase (MAT) enzymes, referred to as SAM synthetases (Figure 2) [76,77]. SAM is required for transmethylation reactions of RNA, DNA and proteins, as illustrated in Figure 2, but also for polyamine and glutathione biosynthesis [76,78,79,80]. Because of the pivotal position of methionine in the metabolic network of the cell, the consequences of methionine availability for cells and organisms are far-reaching, with many mechanisms and signaling pathways feeding into and contributing to the tight control of physiological SAM levels [78,80]. The purpose of this review is to highlight one particular aspect of SAM regulation, which controls gene expression in response to metabolic fluctuations through the regulation of RNA metabolism. 

A strategy commonly used by cells to sense and respond to actual methionine levels is to monitor SAM levels [80]. SAM synthetases, which are highly conserved from prokaryotes to humans, lie at the heart of this regulation [81,82]. Mammalian systems have two distinct catalytic subunits, MAT1A and MAT2A [78,83,84]. MAT1A is mainly found in hepatocytes, whereas MAT2A is ubiquitously expressed and is complexed with a regulatory subunit, MAT2B [78]. Early studies found that the amount of MAT2A is inversely correlated with the cellular methionine concentration, where a decrease in methionine causes an increase in the amount of MAT2A [85,86,87]. Transcriptional and post-transcriptional mechanisms were reported to effect MAT2A levels in the liver [78,81,83,87,88]. Interestingly, both eukaryotes and prokaryotes exploit RNA regulatory elements located within the MAT transcripts to control their expression in response to the need for SAM. RNA structures in SAM synthetases of prokaryotes and fission yeast bind the metabolite directly [89,90]. Bacterial riboswitches represent the paradigm for this concept [89,90]. Here, the direct binding of SAM induces a structural switch in the RNA, which results in the inhibition of transcription and/or translation [89,91,92]. In contrast, in higher eukaryotes, SAM indirectly regulates mRNA levels of SAM synthetase genes. For example, the RNA-binding protein HuR is involved in MAT2A RNA stability control in liver cells [81]. Mechanistically, methylated HuR causes MAT2A mRNA decay [81]. Our understanding of how the regulation of MAT synthetase transcripts can be achieved in other cell types received a boost when recent studies brought a new player into focus: the m^6^A methyltransferase METTL16 [47,54]. It provides an elegant mechanistic explanation of how nutrients and metabolic conditions, previously acknowledged to influence the epigenetic status of a cell, can impact gene regulation via the epitranscriptome in a highly integrated process. 

### 7.2. METTL16 in Mammals Governs SAM Synthetase

**Figure 3:**
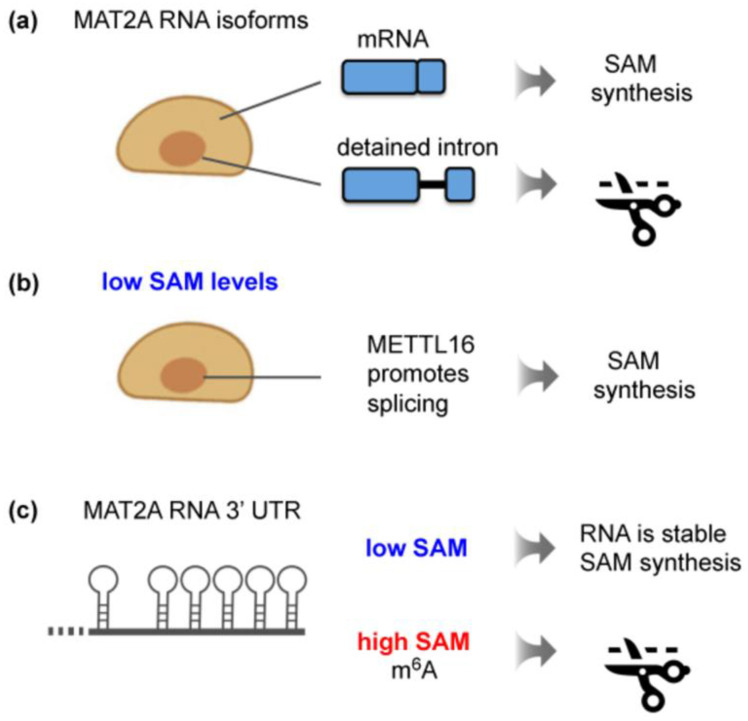
Regulation of mammalian MAT2A expression by METTL16 in response to intracellular SAM levels. (**a**) Mammalian MAT2A has two transcript isoforms: a cytoplasmic mRNA and a nuclear, unproductive isoform with a detained last intron. (**b**) Stable METTL16 binding to the 3′UTR of MAT2A induces full splicing of MAT2A pre-mRNA, leading to increased SAM synthesis. This effect is independent of m^6^A deposition by METTL16. (**c**) The 3′UTR of MAT2A contains six hairpins. Their m^6^A methylation by METTL16 results in SAM-responsive RNA degradation of MAT2A mRNA. Given that the 3′UTR hairpins of MAT2A mRNA are found exclusively in vertebrates [93], a different mechanism must operate in lower eukaryotes.

Six METTL16 consensus methylation sites are present in the 3′ untranslated region (UTR) of MAT2A mRNA, each one positioned in a stem loop and validated as a METTL16 substrate (Figure 3c, left) [47,48,49,93]. These hairpin structures are vital for ensuring the optimal production of SAM synthetase in response to changing SAM levels. The recruitment of METTL16 to these hairpins triggers two distinct events. The first entails the modulation of the MAT2A pre-mRNA splicing pattern in an m^6^A-independent way. The other m^6^A-dependent mechanism involves the regulation of MAT2A mRNA stability. Remarkably, SAM levels determine the precise METTL16 function as described in detail below. 

Extensive studies of MAT2A in mammalian cells have reported the existence of a nuclear transcript isoform that is incompletely spliced [47,86]. This detained-intron MAT2A transcript is subject to nuclear degradation (Figure 3a). A series of elegant experiments from the group of N. Conrad uncovered that, when the SAM supply becomes limiting, METTL16 binding to the 3′UTR enhances the efficiency of co-transcriptional splicing (Figure 3b). This shifts the balance towards the production of mature MAT2A mRNA, ultimately increasing the production of MAT2A protein [47,94]. Intriguingly, it was demonstrated that METTL16 enzymatic activity itself is not required for the induction of MAT2A splicing [47]. In fact, catalytically dead METTL16 promotes the splicing of a reporter MAT2A construct irrespective of the SAM concentration [28]. Here, a single amino acid exchange (N184A) in the SAM-binding site abrogates the methyltransferase activity but retains the RNA-binding capacity of METTL16 [28]. A hyperactive enzyme (K163A), on the other hand, does not enhance splicing [28]. The critical parameter is arguably the dwell time of METTL16 on the 3′UTR, although direct biophysical measurements have not been provided. Low SAM levels were proposed to lead to a decreased enzymatic turnover of METTL16, resulting in an increased residence time [47]. The prolonged binding of METTL16, especially to the hairpin proximal to the intron, stimulates the efficient splicing of MAT2A transcripts. Consistent with this notion, a screen for factors required to induce the splicing of a synthetic MAT2A reporter construct upon methionine depletion identified METTL16. Besides METTL16, other hits were the co-activator of RNA polymerase II MED9 and the cleavage factor I subunit CFIm25 (NUTDT21) [95]. From recent studies, CFIm25 has emerged as a regulator of several RNA-processing events other than polyadenylation through preferential binding to UGU-containing sequences in RNAs [96]. Tethering assays suggest that the association of the CFIm complex with the detained intron and the 3′UTR drives MAT2A splicing [95]. This occurs downstream of METTL16 binding, but how these two events are linked is not understood. Notably, splicing regulation relies on an intact VCR domain of METTL16 [47]. Given that this domain associates with RNA, it was speculated that prolonged METTL16 binding may trigger an RNA conformational change that exposes binding sites for splicing factors [44,47].

Although there is no documented role for m^6^A in the stimulation of MAT2A splicing in mammals, the methylation function of METTL16 is crucial for controlling the steady-state levels of mature MAT2A mRNA (Figure 3c) [28,47,54]. The following findings reveal m^6^A methylation as an integral step in SAM regulation in mammals. When SAM is abundant, an increased METTL16-induced methylation of the hairpins located in the 3′UTR occurs, triggering the destabilization and degradation of the transcript [47,54]. Conversely, an increase in MAT2A mRNA levels observed upon methionine depletion correlates with low m^6^A levels in the 3′UTR of MAT2A [47,54,85]. Consistent with this mechanism of action, catalytically inactive METTL16 causes MAT2A mRNA stabilization, whereas hyperactive METTL16 results in reduced MAT2A mRNA levels [28]. It is not yet clear how METTL16-methylated MAT2A is recognized for degradation and whether known m^6^A reader proteins are involved. Based on experiments with reporter constructs, components of the general m^6^A machinery, such as the reader YTHDC1 and the demethylase FTO, have been implicated in MAT2A mRNA stability control, but understanding the mechanisms requires further exploration [54]. In general, the methylation of MAT2A by METTL16 provides a notable example of an RNA modification that is established in response to a metabolic cue and, in turn, regulates the expression of the target transcript. 

Since mammalian METTL16 can directly influence the methyl-donor capacity of the cell, one would anticipate that the depletion of METTL16 impacts the methylome and transcriptome. Accordingly, it was reported that the *Mettl16* gene is essential for the survival of the vast majority of human cancer cells, for mouse development and in adult mice [11,31,56]. It has proved impossible to bring viable *Mettl16* or *Mat2a* null mice to term [31,56,97]. Knock-in mouse mutants further revealed that both the catalytic activity and the RNA-binding capacity of METTL16 are essential for development [31,56]. The time of death of *Mettl16* knockout embryos is around implantation [31]. Strikingly, at embryonic day 2.5 (E2.5) at the morula stage, embryos that lack *Mettl16* show little alteration in their gene expression, one exception being the *Mat2a* transcript, which is significantly downregulated. This picture changes dramatically in E3.5 blastocysts, which show massive transcriptome dysregulation accompanied by developmental arrest. These data lead to the conclusion that METTL16 activity is essential for embryonic development through the regulation of *Mat2a* mRNA levels and thus SAM availability [31]. 

At this stage, our understanding of the consequence of METTL16 deficiency on the epigenome is limited. One can only speculate that the main cause of the death of *Mettl16* null mice could be either the dysregulation of a single, major event or the sum of several disturbances of metabolic and epigenetic pathways crucial for normal development. Due to the complexity of the processes involved, probing and interpreting the causalities is challenging, but chromatin dynamics may represent a suitable starting point for further interrogation. DNA methylation is undoubtedly the best-understood epigenetic modification in the early development and inactivation of major components of the DNA methylation machinery results in embryonic lethality [98]. At the blastocyst stage, the bulk of genomic DNA is hypomethylated, with the exception of imprinted genes and retrotransposons, whose methylation is maintained [98]. As the embryo implants in the uterus, DNA methylation is widely re-instated [98]. It is possible that METTL16 is involved in this extensive DNA methylation reprogramming. Therefore, it will be exciting to directly investigate whether DNA methylation is one of the processes that become disrupted in the *Mettl16* mutants and whether it is maintenance and/or de-novo methylation, which is deficient. Histone methylation may also be affected, although pilot experiments in human cells lacking METTL16 have not provided supporting evidence [11]. A future systematic genome-wide interrogation of the potential impact on histone and DNA methylation in human and mouse cells lacking METTL16 should provide answers to the question of whether fluctuations in SAM levels in response to a loss of METTL16 are sufficient to alter the epigenetic landscape. 

Collectively, these studies in mammals postulate that METTL16 can act as an SAM sensor, but the generality of this is not clear. It is therefore important to turn to other model organisms such as invertebrates and further interrogate the relevance of the catalytic activity of METTL16.

### 7.3. METTL16 in Nematodes Regulates SAM Synthetase Pre-mRNA Splicing via m^6^A

It turns out that SAM production in nematodes is also modulated by METTL16 [56,99]. This involves m^6^A methylation catalyzed by METTL16, which, in turn, affects pre-mRNA splicing. It is worth stressing that METTL16 impacts MAT2A splicing in different ways in mammals and in worms. One, the splice events occur at different locations in the MAT2A transcript. Two, the splicing of mammalian MAT2A pre-mRNA involves METTL16 binding but not methylation. Therefore, m^6^A has no apparent role in this particular splicing phenotype in mammals. In contrast, m^6^A is central to the MAT2A-splicing phenotype in worms. The mechanistic details were uncovered by taking advantage of the fact that nematodes lack the METTL3/14 m^6^A writer complex dominant in mammals [56,100], making them an ideal model to study the molecular and physiological effect of METTL16. The *C. elegans mettl16* ortholog, *mett-10,* is required for normal development [40]. Although the METT-10 enzyme lacks the vertebrate specific C-terminus it methylates similar substrates as its mammalian counterpart, particularly U6 snRNA and *sams-3, sams-4 and sams-5* transcripts, the *C. elegans* orthologs of mammalian *MAT2A* [56,99]. Notably, the number and location of the methylation sites in the SAM synthetase transcripts differ between mammals and worms [56]. This is not surprising since *sams* transcripts do not contain the 3′UTR hairpins conserved in MAT2A mRNAs of vertebrates [93]. Instead, a single m^6^A is present in worm SAM transcripts in a nonamer sequence that closely resembles the mammalian consensus, UACm^6^AGAaAc (lower case indicates worm specific bases), and is predicted to fold into a stem-loop structure. Intriguingly, the modified adenine base within this motif is at a location known to play a major role in splice site selection: at the invariant AG dinucleotide at the 3′ end of an intron. Two landmark studies uncovered that the methylation of this adenosine residue inhibits the use of this particular splice site, ultimately controlling the steady-state level of SAM synthetase [56,99]. How is this achieved given that the *C. elegans* genome does not code for orthologs of the YTH family of m^6^A readers or demethylases [100]? It was shown that the methyl-mark set by METT-10 precludes the binding of the essential splicing factor U2AF35, preventing spliceosome assembly [56,99,101]. As a consequence, alternative splicing coupled with nonsense-mediated mRNA decay occurs, leading to reduced levels of the SAM synthetase [56,99]. This model was developed to explain observations of m^6^A immunoprecipitates being enriched in the non-productive *sams* isoforms but depleted for the correctly spliced, productive mRNA [99]. On the other hand, an increase in the correctly spliced, productive *sams* isoform was observed in *mett-10* mutant worms [56,99]. Using transgenic worms, it was further confirmed that exonic mutations that abolish 3′ slice site m^6^A methylation in vitro allow for efficient splicing in vivo [56]. 

A link between m^6^A and pre-mRNA splicing has been appreciated for years in many model organisms, especially in *Drosophila* [19]. One challenge in mammals has been to distinguish between direct versus indirect roles of m^6^A in splicing control. The described studies in worms provide the first demonstration that the presence of an m^6^A modification at a 3′splice site can directly interfere with splicing. Splice sites and the mechanisms of their recognition are highly conserved across the animal kingdom. This prompted Mendel et al. [56] to investigate whether the human splicing machinery is similarly sensitive to the presence of an m^6^A. In a series of elegant experiments, they artificially introduced an m^6^A either at a 3′ splice site or into an unrelated exon sequence of a human reporter construct and performed splicing assays in HeLa cell extracts. Exonic methylation did not inhibit splicing whereas methylation of the 3′ splice site did. Using a computational approach, putative 3′ splice site targets for mammalian METTL16 were identified. While mouse METTL16 was found to have the potential to methylate these 3′ splice sites in vitro, data on the in vivo significance are missing [56]. The jury is thus still out as to whether alternative splicing control by means of m^6^A methylation of an 3′splice site is the conserved principal mechanism beyond invertebrates. Interestingly, intronic polyadenylation has recently been linked to METTL16 methylation activity in mammals [51], suggesting that m^6^A deposited by METTL16 can determine the choice of alternative pre-mRNA-processing events in various ways. 

Crucially, methylation and the alternative splicing of nematode SAM synthetase transcripts are linked to nutrient levels [56,99], highlighting the capacity of METT-10 to sense and respond to nutrient availability. In response to high SAM levels in nutrient-rich media, METT-10 installs m^6^A at a splice site to inhibit productive splicing and hence SAM synthetase production. Under low-nutrient conditions, m^6^A is absent, allowing for efficient splicing and the production of functional SAM synthetase, which, in turn, can generate more SAM from methionine and ATP. Accordingly, SAM synthetase activity autoregulates the expression of SAM synthetase genes in worms in response to nutrient availability through alternative splicing involving METT-10 enzyme activity. To understand the functional implications, mutants that interfere with the *mett-10* function and SAM synthesis were analyzed and shown to have fertility defects [56]. What is missing is information on whether epigenetic pathways are disrupted in *mett-10* mutant worms. *C. elegans* lacks DNA methylation but it has been shown that the depletion of sams-3 and sams-4 globally reduces histone methylation and disrupts heterochromatin organization [102]. This demonstrates that normal SAM levels are critical for maintaining the *C. elegans* epigenome and implies that m^6^A methylation deposited by METTL16 could play a significant part in this regulation.

How conserved is this mechanism of controlling cellular SAM levels via alternative splicing of the SAM synthetase pre-mRNAs in other invertebrates? At the center of this regulation lies a nonamer sequence that is recognized by METT-10 when imbedded within a stem loop structure at an exon-intron border and methylated in response to a rich diet [56,99]. Based on the fact that this sequence is conserved among worms, silk moth and flies and can indeed be methylated by METTL16 in vitro [56] it has been proposed that this type of SAM synthetase regulation may be widely used among invertebrates. 

### 7.4. METTL16 in Plants and Fission Yeast Has Not Been Implicated in SAM Homeostasis

Because MAT2A is an established target of METTL16 in the vertebrates and invertebrates studied thus far, the question pertains as to whether other species use METTL16 to regulate SAM synthetase expression. METTL16 is highly conserved across many metazoans, as well as in plants, fission yeast and bacteria [31,44,47,55]. Of these representative species, METTL16 has been functionally characterized in mammals, *C. elegans*, *Arabidopsis thaliana* and fission yeast. *S. pombe* possesses a single gene for SAM synthetase, *sam1*. The fission yeast METTL16, Mtl16, neither methylates SAM synthetase RNA nor does its deletion affect the transcript levels of *sam1* [71]. The *sam1* transcript harbors a tertiary structure in its 5′UTR, which, upon binding directly to the SAM molecule, mediates repression of translation [90]. This is analogous to the ligand-sensing *sam1* mRNA in bacteria, which regulates the SAM metabolism in a negative feedback cycle [89]. 

The *Arabidopsis* METTL16 ortholog is called FIONA1 (encoded by *At2g21070*). Originally described as a regulator of circadian rhythms [103], FIONA1 was very recently shown to be a bona fide RNA methyltransferase [69,70]. Plants, unlike worms, additionally contain the m^6^A methyltransferase Mettl3/14 enzyme complex, termed MTA (encoded by *At4g10760*) and MTB (encoded by *At4g09980*), that is responsible for the vast majority of m^6^A methylation [104,105]. Knockout mutations in the MTA/MTB genes are embryonic lethal in *Arabidopsis* [59,60], underscoring the importance for m^6^A RNA methylation in plant development. The disruption of FIONA1, on the other hand, results in early flowering and, at the molecular level, in a mild decrease (10–15%) in global m^6^A levels, which, in turn, can be restored by the expression of active FIONA1 [69,70]. The U6 splicing snRNA, as well as a small subset of mRNAs, have been identified as FIONA1-specific m^6^A target sites [69,70]. The functional consequences of FIONA1-dependent methylation involve the regulation of transcript abundance and alternative polyadenylation [69,70]. 

SAM deficiency suppresses the methylation of DNA and histones in rice, leading to a late-flowering phenotype [106]. However, how SAM synthetase activity is controlled is not understood as the regulatory subunit MAT2B is lacking in plants [107]. The MAT2A catalytic subunit is encoded by the *MAT1-4* genes in *Arabidopsis* [107]. The corresponding transcripts have detectable m^6^A methylation but whether it is installed by FIONA1 is controversial; two groups disagree on whether methyl marks in *MAT1-4* are reduced when FIONA1 function is disrupted [69,70]. The reasons for this discrepancy could be technical, given that seedlings of different ages were investigated by different methods: either m^6^A- or nanopore sequencing. Nevertheless, the consensus from both studies is that the expression levels of *MAT1-4* transcripts are not affected by FIONA1, neither under normal nor high-SAM conditions [69,70]. Whether FIONA1 regulates *MAT* transcript processing in different ways—for instance, at the level of localization or translation—has not been investigated. FIONA1 is not essential for viability [69], which may argue against this enzyme being a key modulator of SAM levels. On balance, the evidence to date does not provide strong support for the role of FIONA1 in the regulation of SAM homeostasis. It remains to be seen whether other RNA methyltransferases contribute to the control of SAM synthetase activity in plants or what alternative mechanisms exist.

## 8. Concluding Remarks

METTL16 is a versatile RNA-binding and modifying enzyme engaged in the control of splice site selection, RNA stability and translation [11,31,47,54,56,58,71,95]. Through its multlifunctionality it influences various cellular processes, including the maintenance of genome integrity, proliferation, erythropoiesis and cancer progression [11,52,53,57,59,80,108]. Future efforts to understand these diverse roles will benefit from the identification of METTL16 regulators and effectors that, so far, have remained largely elusive. One of the best-understood and fundamental functions of METTL16 is the fine-tuning of MAT2A expression to regulate cellular SAM levels in humans, mice and nematodes [28,31,47,56,94,99]. To achieve this, METTL16 drives self-sustaining feedback loops that link MAT2A transcript abundance with SAM synthesis. It will now be important to investigate METTL16 function in MAT2A regulation in pluripotent stem cells and cancer models, as these cell types have an unusually high dependence on methionine, and altered metabolism is a hallmark of cancer [79,109,110,111,112,113]. Work in human and animal models has documented that changes in SAM levels can impact chromatin organization and actively contribute to the regulation of transcriptional programs [109,110,114,115,116,117]. To what extent the METTL16-dependent regulation of SAM synthesis correlates with epigenetic changes in DNA and histone methylation and under which circumstances it impacts chromatin states remains to be determined. 

The physiological strategy to utilize RNAs for the regulation of SAM homeostasis is interesting in light of the fact that RNA transcripts turn over and RNA modifications will be lost upon turnover. This may allow for a swift yet relatively transient and therefore flexible response to changing environmental conditions. The involvement of multiple METTL16 target stem loops in MAT2A regulation in mammals is particularly intriguing as it suggests a co-operative mechanism for sensing SAM levels [54] rather than a simple on and off switch. One could envision a model in which the number of sites methylated by METTL16 at any one time allows for fine control, like a dimmer switch. Structural studies are in line with this idea, indicating that different hairpins are methylated with varying efficiencies when using pure enzymes [28]. Such a cooperative mechanism would provide a sophisticated regulatory response to fluctuating SAM levels.

Overall, the results reviewed here draw attention to METTL16 as a paradigm of an RNA methyltransferase at the intersection of metabolism and gene regulation. One principle that has emerged is that, in mammals and worms, METTL16/METT-10-mediated methylation events turn SAM production down in response to high intracellular SAM levels. In mammals, the regulation is more sophisticated, with METTL16 additionally ensuring an increase in SAM production when the demand rises. The mechanistic detail is revealing because, in the absence of SAM, METTL16 binds stably to unmodified MAT2A RNA and performs a non-catalytic function. Therefore, METTL16 can be considered as a writer and a reader. These distinct METTL16-dependent layers of SAM regulation observed in mammals are reminiscent of belt and braces and likely part of an intricate, multi-layered regulatory network preserving physiological SAM levels. An added complexity arises from the fact that SAM is interconnected with other metabolic pathways that affect nearly all aspects of cellular physiology [78,80], making it challenging to demonstrate causality. At the same time, this opens many avenues for further exploration and will likely drive the development of new concepts that will advance our understanding of the interplay between chromatin, RNA and metabolic networks in genome regulation.

## Figures and Tables

**Table 1:** Specificity of m^6^A writers. Modified residue is underlined.

m^6^A MTFase	Catalytic Activity	Validated m^6^A Targets	Substrate Specificity	KO Mice
METTL3 METTL14	yes no	mRNAs ncRNAs microRNAs	DRACH	lethal
METTL16	yes	MAT2A mRNA U6 snRNA	UACAGAGAA in stem loop	lethal
METTL5	yes	18S rRNA	UAACA	viable
ZCCHC4	yes	28S rRNA	AAC in stem loop	viable

Ref.: METTL3/14 [5,20,27,29,30] METTL16 [28,31] METTL5 [32,33,34] ZCCHC4 [26,35,36].
